# *SUePDF*: a program to obtain quantitative pair distribution functions from electron diffraction data

**DOI:** 10.1107/S160057671601863X

**Published:** 2017-02-01

**Authors:** Dung Trung Tran, Gunnar Svensson, Cheuk-Wai Tai

**Affiliations:** a Department of Materials and Environmental Chemistry, Arrhenius Laboratory, Stockholm University, Svante Arrhenius väg 16C, Stockholm, S-10691, Sweden

**Keywords:** pair distribution functions, electron diffraction, transmission electron microscopy, disorder

## Abstract

*SUePDF* is a graphical user interface program written in MATLAB to achieve quantitative pair distribution functions (PDFs) from electron diffraction data. The program facilitates structural studies of amorphous materials and small nanoparticles using electron diffraction data from transmission electron microscopes. It is based on the physics of electron scattering as well as the total scattering methodology. A method of background modeling is introduced to treat the intensity tail of the direct beam, inelastic scattering and incoherent multiple scattering. Kinematical electron scattering intensity is scaled using the electron scattering factors. The PDFs obtained after Fourier transforms are normalized with respect to number density, nanoparticle form factor and the non-negativity of probability density. *SUePDF* is distributed as free software for academic users.

## Abstract 2

*SUePDF* is software to convert electron diffraction data to quantitative pair distribution functions.

## 1. Introduction   

The pair distribution function (PDF) method is widely employed for studying structurally disordered materials (Warren, 1990 ▸; Egami & Billinge, 2002 ▸; Proffen *et al.*, 2003 ▸). This is because it gives more structural information, beyond the information given by the traditional Bragg-peak-based analysis, from scattering data. Standard computer programs for PDFs for neutron and X-ray powder diffraction data are well established (Peterson *et al.*, 2000 ▸; Juhás *et al.*, 2013 ▸). A number of efforts have been made to obtain PDFs from electron diffraction (ED) data (Cockayne & McKenzie, 1988 ▸; Tewes *et al.*, 1994 ▸; Takagi *et al.*, 2001 ▸; McBride, 2003 ▸; Ankele *et al.*, 2005 ▸; Ishimaru *et al.*, 2008 ▸; Abeykoon *et al.*, 2012 ▸; Mu *et al.*, 2013 ▸). However, these have not been sufficient to establish electron powder diffraction as one of the major data sources for PDF analysis. The main obstacle has been the multiple scattering of electrons, which may alter the scattering intensities in the ED patterns, making it difficult to extract an undistorted structure function (Uyeda, 1968 ▸; Cowley, 1969 ▸; Anstis *et al.*, 1988 ▸; Cockayne & McKenzie, 1988 ▸). Beside this, the electron–matter interaction differs from the cases of X-rays and neutrons, implying that a dedicated procedure of data treatment is needed for ED data.

In order to obtain reasonable PDFs, ED data 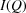 have to be scaled using appropriate electron scattering factors 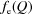 to yield a proper reduced structure function 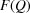, where *Q* is the magnitude of the scattering vector. This is not an easy task because the ED data are generally contaminated by inelastic scattering and distorted by multiple scattering, instrument errors and noise. The widely reported solution for inelastic scattering is using an energy filter, but this may also cause additional distortion in the data when only electrons of certain energy-loss range can be blocked. Data scaling was previously carried out by multiplying the compositional averages 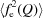 and 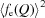 with a fitting parameter η, which may be fixed by matching 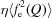 with 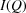 at large *Q* (Cockayne & McKenzie, 1988 ▸; Cockcayne, 2007 ▸; Cockayne *et al.*, 2010 ▸). An early software attempt to fit 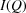 with 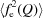 for PDF extraction was made by drawing and then subtracting a background of 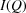 (Hauschild, 2009 ▸). However, because the mathematical curve presented for this background is generally not suitable, the background is often constructed manually. The next software attempt was made by Mitchell & Petersen (2012 ▸), in which an additional fitting parameter α was introduced to counter the discrepancy between 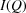 and 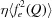. This meant that 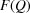 was obtained by matching 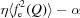 with 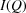 at large *Q*. In other work, instead of introducing an additional parameter, 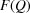 was directly adjusted by fitting the distortion feature to a fourth-order polynomial (Mu *et al.*, 2013 ▸).

The program *SUePDF* aims to effectively correct ED data by taking into account the multiple scattering features and uniqueness of electron–matter interactions. For amorphous materials, the coherent multiple scattering can be considered as insignificant and incoherent multiple scattering should merely contribute to the background (Cowley, 1992 ▸), which may be modeled and subtracted. For crystalline-like nanoparticles, coherent multiple scattering should not affect the peak positions of the resulting PDFs but only the peak intensities. This has been demonstrated to be the case for crystals having a thickness of less than five times the electron mean free path (Anstis *et al.*, 1988 ▸). The problem with modulated peak intensities caused by coherent multiple scattering is tackled in *SUePDF* by a renormalization procedure based on number densities, and the probability of having an atom at a certain distance is non-negative. In general, the major distortion of 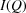 can be considered to form a smooth background, which is built up from the direct-beam tail, inelastic continuum and incoherent multiple scattering. *SUePDF* employs the combination of an optimizing parameterization and a reasonable mathematical model for the background. Inside *SUePDF*, background subtraction is coupled with data scaling in a loop-based routine to optimize the data treatment. After background subtraction and data scaling, the PDF can be obtained by a Fourier transform of the normalized data. On the basis of the physical meaning of PDFs, noise filtering and normalization procedures have to be routinely carried out. This improves the physical reliability of the outcome PDFs and allows the uncertainties to be evaluated. *SUePDF* also offers the possibility to correct finite size effects present for nanoparticles, by using a nanoparticle form factor computed for a given size and shape.

## 2. Methods   

### 2.1. Background modeling   

**Figure 1:**
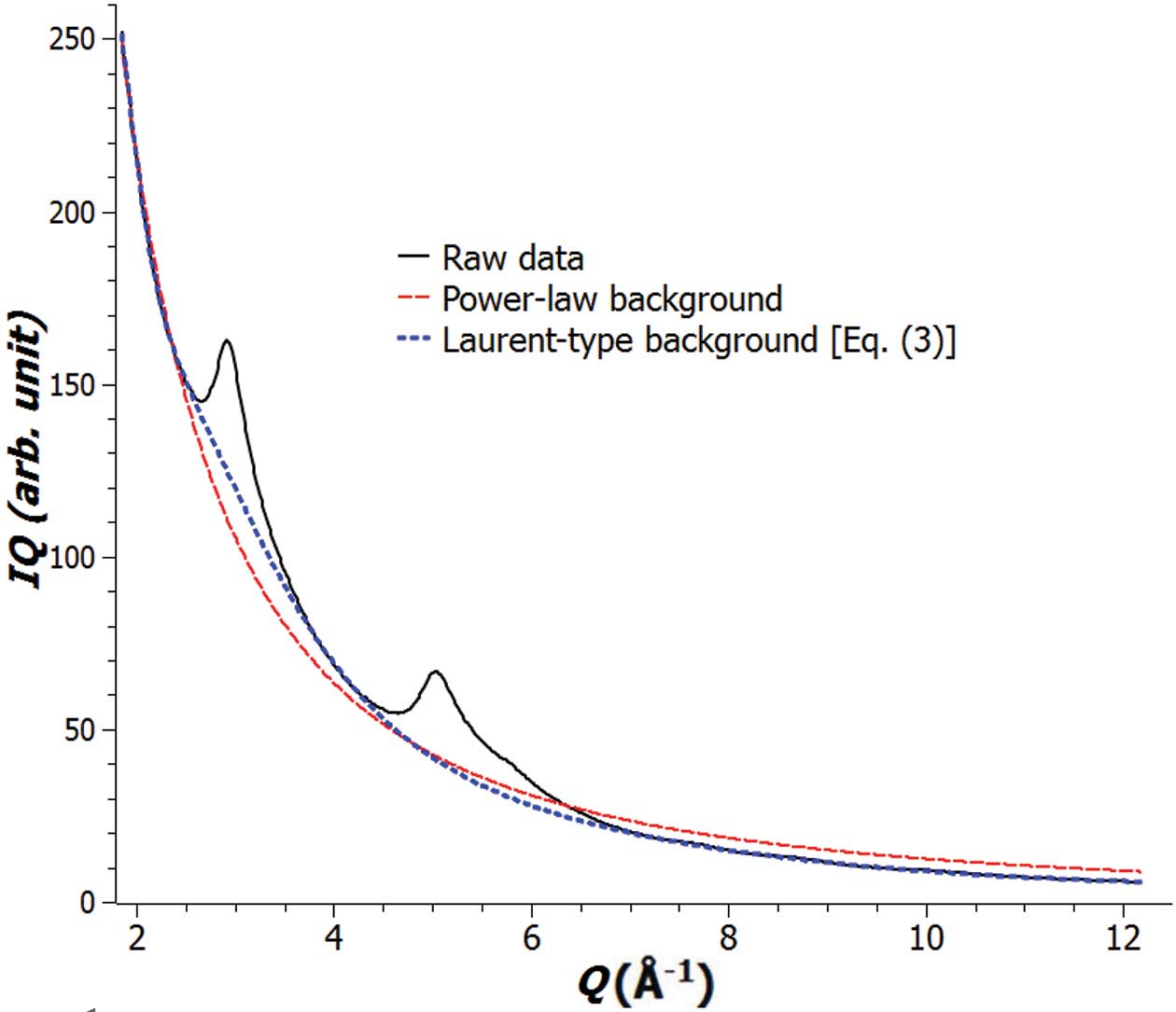
Background modeling for electron powder diffraction data (solid black line) of nanoporous carbon: power-law model (dashed red line) compared with Laurent-type model (dotted blue line) [equation (3)] with 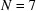.

The smooth background observed for ED data is considered to be the contributions from the direct-beam tail, inelastic scattering continuum and incoherent multiple scattering. For an elastic scattering of a λ-wavelength electron with a semi-angle θ, 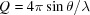, but in the case of small-angle inelastic scattering (Egerton, 2011 ▸)
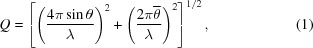
where 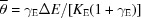 is the characteristic angle corresponding to an energy loss 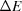; 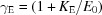 is the relativistic factor with 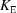 the kinetic energy and 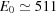
 keV the stationary mass-converted energy of the electron. Equation (1) can be rewritten for the inelastic component when 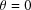 as
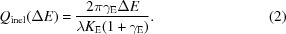
For example, if the electron energy loss is counted up to 2000 eV for an incident beam of 200 keV, then the inelastic beam tail is limited to below 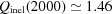
 Å^−1^. For low-loss electrons (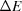 < 50 eV), the inelastic component 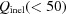 is less than ∼0.036 Å^−1^. Equation (2) means that the inelastic error is more significant for electrons of high energy loss. It is well known that high-loss electrons can be excluded using an energy filter. However, according to our experience, energy filtering also modifies the background, making an accurate quantitative background modeling difficult. The following mathematical model is introduced to fit the background 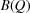 of the electron powder diffraction pattern: 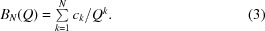
Equation (3) is actually a positive-degree portion of a Laurent-type series where 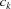 are the fitting parameters and *N* is the fitting order, which may vary for different sample compositions. A comparison between the power-law model and the Laurent-type model [described in equation (3), with 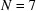] for background fitting of electron powder diffraction data acquired from a nanoporous carbon sample is shown in Fig. 1 ▸. In addition, this background model works in both thin and thick samples. This is demonstrated in a study of amorphous silica discussed in §[Sec sec4.2]4.2. The measured bond lengths and the values reported in the literature are listed there and are in good agreement.

### 2.2. Optimization procedure for data scaling and background modeling   

Electron scattering factors, denoted as 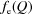, are used for data scaling. These factors may be obtained using the following Mott–Bethe formula (Mott & Massey, 1965 ▸): 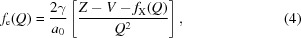
where *Z* is the atomic number, *V* is the valence number, 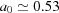
 Å is the Bohr radius and 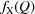 is the X-ray scattering factor (Brown *et al.*, 2006 ▸). For neutral atoms, it is recommended to use the available DFT-computation-based parameterizations (Kirkland, 2010 ▸), which are considered to be more accurate than those from the Mott–Bethe formula, particularly at low *Q* values. For samples composed of more than one element, the chemical compositions are required as input, together with the corresponding valence for each element.

**Figure 2:**
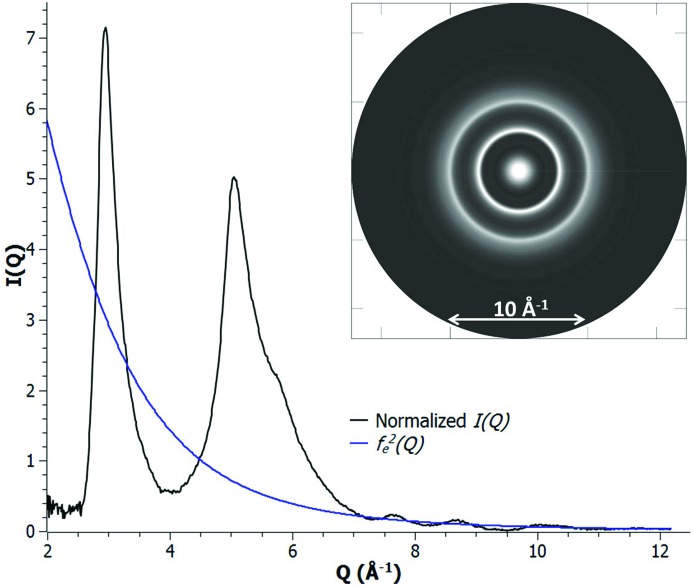
Normalized 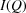 of a nanoporous carbon sample (back solid) and the corresponding 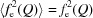; the inset shows a ring pattern reconstructed from this normalized 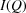.

The data scaling is constrained by a mathematical feature of the structure function 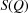. That is, 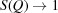 when 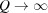. The background-subtracted and normalized scattering intensity 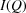 is converted into 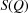 by using the composition-averaged 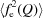 and 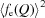 (Warren, 1990 ▸): 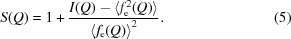
The data scaling is done according to
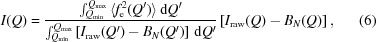
where 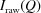 is the raw data of scattering intensity. The feature 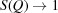 when 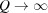 restricts 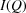 to attenuate around 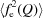 at high *Q* values. This suggests a necessary minimization of the following quantity, defined at the tail of 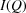: 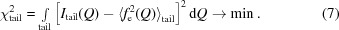
In *SUePDF*, the minimization of equation (7) is carried out in a loop procedure by varying the background reference and the fitting order *N*, which are initially input by users, to optimize the normalized 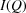. A normalized 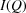 of a nanoporous carbon sample, scaled by the corresponding 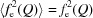, is shown in Fig. 2 ▸.

### 2.3. Fourier transform of *F*(*Q*) to yield reduced PDF   

**Figure 3:**
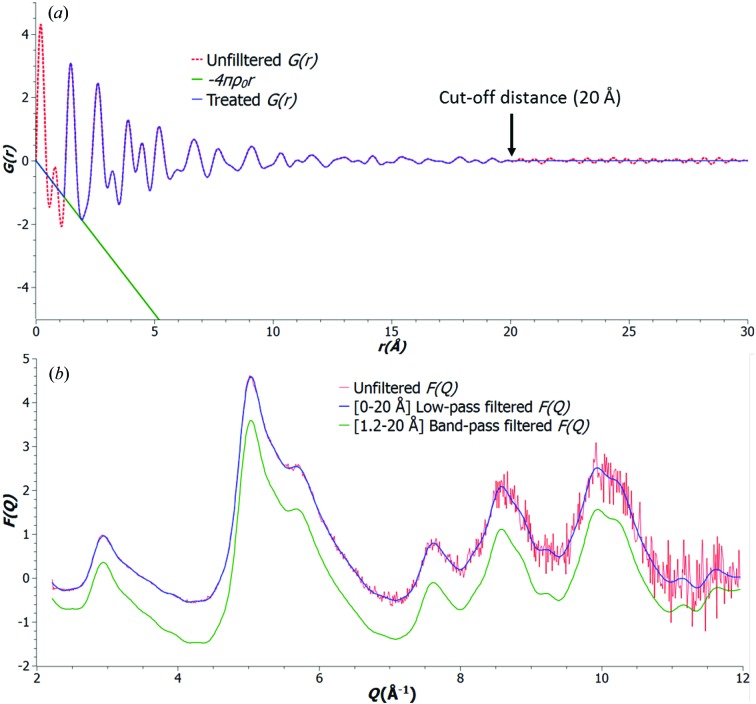
(*a*) Normalization and noise treatment for 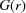 of nanoporous carbon, with 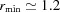
 Å and 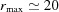
 Å; (*b*) [0–20 Å] low-pass-filtered 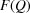 (blue solid line) and [1.2–20 Å] band-pass-filtered 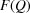 (green solid line) compared with the unfiltered 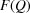 (red).

The reduced PDF 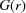 is obtained by a Fourier transform of the reduced structure function 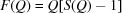: 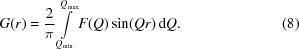
The red dotted curve shown in Fig. 3 ▸(*a*) is the unfiltered 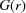 for nanoporous carbon after the Fourier transform [equation (8)]. Besides being convolved with the termination function 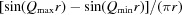 (Peterson *et al.*, 2003 ▸), this unfiltered 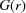 exhibits some artifact peaks at low (<1 Å) and high (>20 Å) values of *r*, which are equivalent to the low- and high-frequency noise, respectively, of the experimental data.

### 2.4. PDF normalization and noise filtering   

The unfiltered 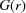 obtained from equation (8) has to be adjusted by physical and mathematical constraints. These constraints refer back to the definition of PDF 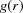, which represents the probability density of finding a pair of two atoms separated by distance *r* (Egami & Billinge, 2002 ▸). Therefore 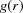, the probability density, has to be non-negative: 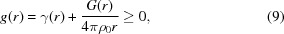
where 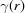 is the nanoparticle form factor (Kodama *et al.*, 2006 ▸; Gilbert, 2008 ▸; Tran *et al.*, 2016 ▸) [for bulk, 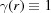] and 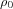 is the average number density of the sample. The normalization of the PDFs is based on equation (9). Prior knowledge about the shortest interatomic distance existing in the sample is used as a physical constraint, such that the PDF 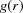 at distances smaller than a value 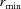 is set to zero: 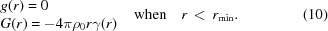
Equation (10) is used to filter off low-frequency noise. The high-frequency noise can be filtered off by setting an upper cut-off distance 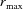, where 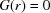 at 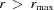. This noise treatment of 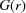 is demonstrated in Fig. 3 ▸(*a*). A Fourier back-transform of the treated 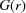 then yields the noise-filtered 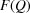 data, shown in Fig. 3 ▸(*b*), for the here given example from a nanoporous carbon sample.

### 2.5. Evaluation of uncertainty   

**Figure 4:**
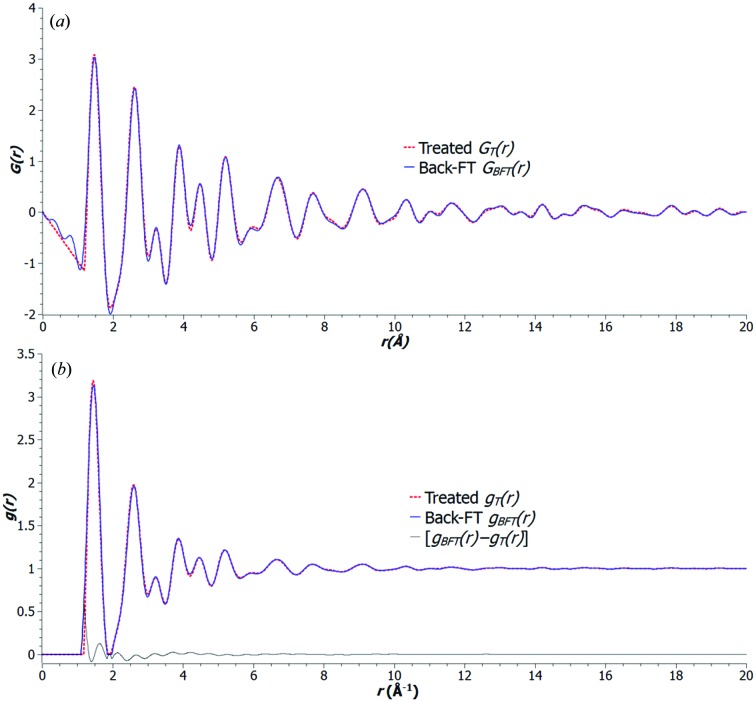
Uncertainty in the noise filtering of PDFs: (*a*) treated 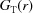 (red dotted line) compared with 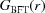 (blue solid line) which has been transformed from [1.2–20 Å] band-pass-filtered 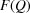; (*b*) the corresponding normalized PDFs 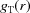 (red dotted line) and 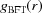 (blue solid line) and the difference between these (black line). From these, the evaluated uncertainty is ∼3.7%.

The treated reduced PDF 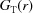 is compared with 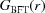, which is the Fourier transform of the [1.2–20 Å] band-pass-filtered 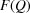, in Fig. 4 ▸(*a*). The observed difference between 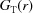 and 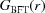 is the consequence of the cutting of low and high frequencies which is propagated through the Fourier transform. Therefore, the uncertainty considered here is mainly associated with the low- and high-frequency noise, which correspond to the artifact short- and long-distance peaks found in the unfiltered 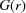, respectively.

From Fig. 3 ▸(*b*), it is clear that the low-frequency noise can be attributed to some low-frequency distortions of the 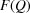 data. These distortions may be caused by the imperfection of the instrumental setup, the errors encountered during the background subtraction and data scaling using the approximated electron scattering factors. One of the typical errors is the beam convergence, which generally does not affect the positions of PDF peaks but does affect their intensities (McBride, 2003 ▸). Beside these errors, in the case of coherent structures, the low-frequency distortions may be related to the coherent multiple scattering which might not be treated properly by background subtraction. The high-frequency noise, which is believed to be more random, may come from various sources. Some probable sources are the electromagnetic environment, mechanical instability of the instrument *etc*. Note that the properties of the recording media are of importance and can influence the results in both images and diffraction significantly, in particular resolution and sensitivity (Ruskin *et al.*, 2013 ▸). In general, the higher the number of pixels in the detector, the better the resolution that can be achieved. The current generation of detectors, which are of CCD and CMOS type and have either 1000 × 1000 or 2000 × 2000 pixels installed in a typical electron microscope configuration, can provide significantly high *Q*_max_ for most applications. A detector with better dynamical range and sensitivity can have the advantage of handling strong diffraction in the low-*Q* range and acquiring a weaker signal in the higher-*Q* range. As a result, the extended *Q* range can improve the overall quality of the ED-based PDF. The optimization of the acquisition condition for a particular detector can help to obtain quality electron diffraction patterns and therefore ED-based PDFs, especially to minimize artifacts given by the detector, such as streaking, the trace of the beam path given by the shutter, blooming *etc*.

The uncertainty of the normalized PDFs can be evaluated from the relative r.m.s. difference between the corresponding 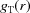 and 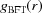: 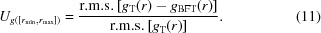

*U*_g([1.2 Å, 20 Å])_ ≃ 3.7% for the case of the nanoporous carbon sample shown in Fig. 4 ▸(*b*).

## 3. The graphical user interface of *SUePDF*   

### 3.1. Electron total scattering profile input   

**Figure 5:**
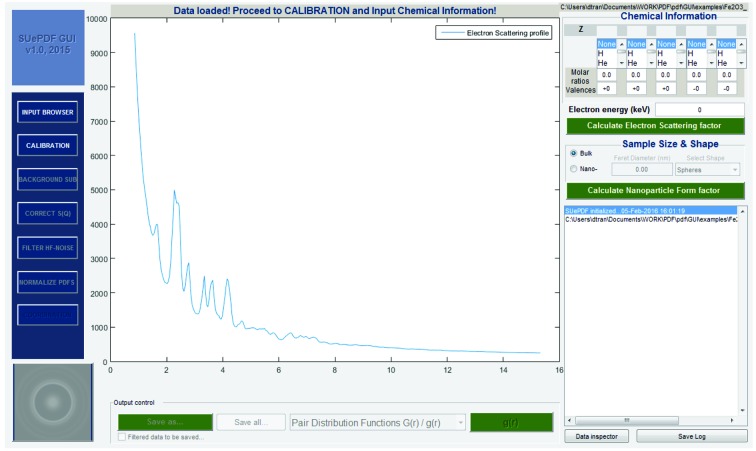
An overview of the *SUePDF* GUI after inputting the data file of an electron scattering profile.

The input data for *SUePDF* v1.0 is a one-dimensional electron total scattering intensity profile. The input file must have a simple *x*–*y* two-column format. The *x* column is the *s* values (in Å^−1^ when calibrated; note that *Q* = 2π*s*) and the *y* column is the corresponding intensity *I*(*Q*). Multiple input files may be selected for integration of different diffraction data of the same sample and under the same experimental conditions. Multiple input files must be synchronized with the same format and the same data size. Fig. 5 ▸ shows an overview of the graphical user interface (GUI) after inputting a data file using ‘INPUT BROWSER’.

### 3.2. Loading electron scattering factors   

**Figure 6:**
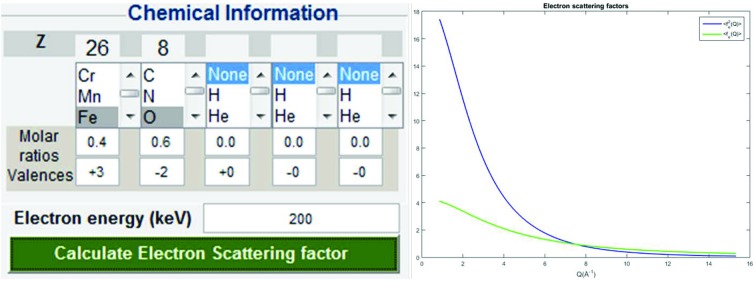
The GUI for calculating the electron scattering factor by inputting chemical information and electron kinetic energy.

Databases of parameterization for both electron (Kirkland, 2010 ▸) and X-ray (Brown *et al.*, 2006 ▸) scattering factors (or atomic form factors) are implemented in *SUePDF*. In cases of neutral atoms the electron database will be used; otherwise the X-ray database will be loaded in order to calculate the electron scattering factors for ions *via* the Mott–Bethe formula [equation (4)]. Besides the electron energy (in keV), chemical information, including the elemental composition, molar ratios and valences, is required as input to load appropriate electron scattering factors for data scaling. Fig. 6 ▸ shows the GUI panel for inputting chemical information and electron kinetic energy and calculation of electron scattering factors.

### 3.3. Background optimization   

The background optimization is based on user input of the following:

(i) Two points specifying the pre-peak background (corresponding to the lowest momentum transfer) and the tail.

(ii) The number of middle background reference points: these points will be automatically positioned as initial conditions between the previous two points of the pre-peak and the tail. Their positions will vary along the curve of the raw scattering profile to optimize the background. It is noted that these reference points generally do not lie on the background; their distances to the background are refined while their positions vary. The typically recommended number of these reference points is 3–8. Use of larger numbers of reference points consumes more computation time.

(iii) The maximum fitting order: typically recommended orders are 5–15. Larger fitting orders consume more computation time.

**Figure 7:**
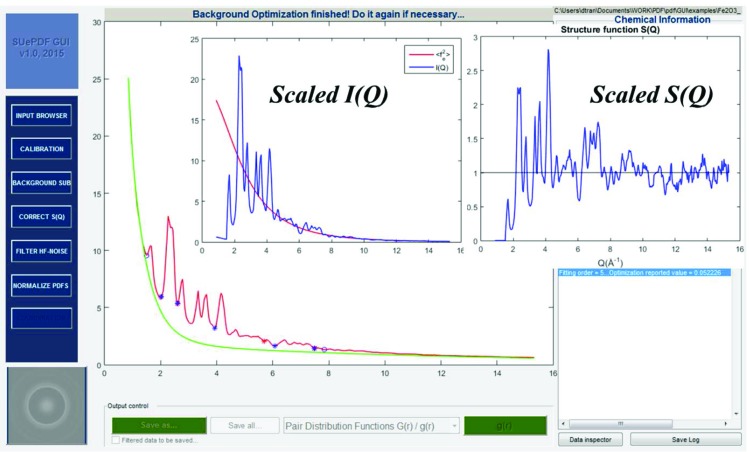
An optimized background and the corresponding scaled *I*(*Q*) and *S*(*Q*).

Fig. 7 ▸ shows a background optimized in the *SUePDF* GUI, together with the scaled *I*(*Q*) and *S*(*Q*).

### 3.4. *S*(*Q*) correction and high-frequency noise filtering   

*SUePDF* offers a routine for correction of the *S*(*Q*) tail. This routine is optional and only recommended when a good enough solution for the *S*(*Q*) scaling problem cannot be found by background optimization. The correction procedure is based on calculation of the median curve of the *S*(*Q*) tail. There are two steps:

(1) Specifying the to-be-corrected tail of *S*(*Q*).

(2) Tuning the order of the median fitting to achieve a corrected tail of *S*(*Q*). The tuning may also be done automatically by pressing the ‘Auto optimization’ button.

High-frequency noise filtering is recommended because ED data usually contain high-frequency noise, which is more visible at high *Q* values as spiky oscillations. The filter is based on forward-and-back Fourier transforms. A cut-off distance is required as a user input. The cut-off distance serves as the ‘highest frequency’ allowed in the ED data and is supposed to relate to the atomic structure.

### 3.5. Nanoparticle form factor   

**Figure 8:**
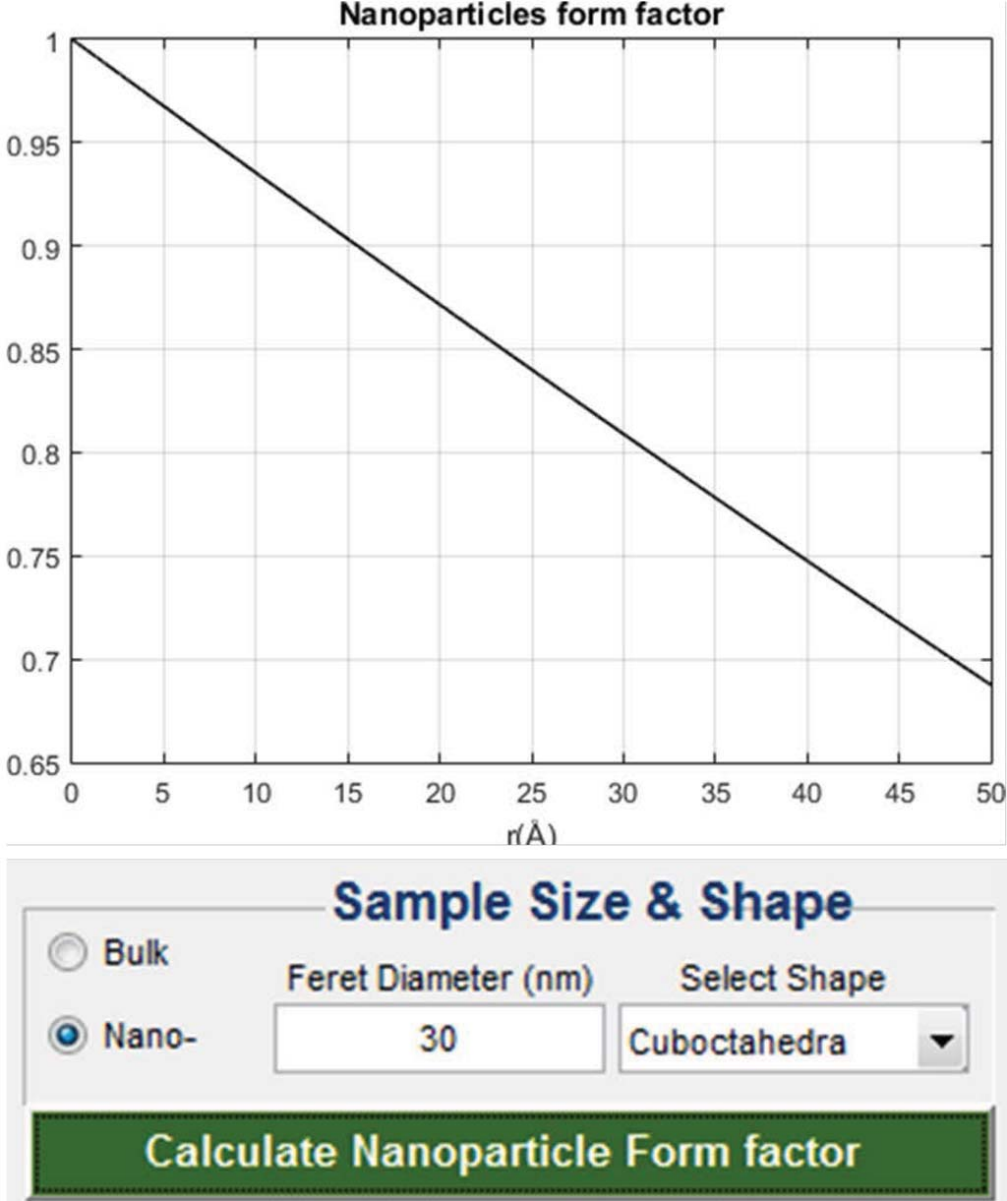
GUI panel for calculation of nanoparticle form factors.

The nanoparticle form factor takes into account the size and shape of the sample and quantifies how much they affect the normalized PDF. For a bulk sample this factor is unity. *SUePDF* v.1.0 offers the calculations for four basic shapes: sphere, cuboctahedron, cube and truncated cube (Tran *et al.*, 2016 ▸). When working with nanoparticle samples, the user must input their size and shape to load the appropriate form factor. If this information is not loaded, the default form factor will be unity (for bulk). Fig. 8 ▸ shows the GUI panel for calculation of the nanoparticle form factor.

### 3.6. PDF renormalization   

This renormalization is to achieve the quantitative PDF *g*(*r*). The renormalization procedure is based on the following:

(*a*) The non-negativity of *g*(*r*) as probability density.

(*b*) The cutting off of short unphysical distances producing low-frequency distortion in the data (low-frequency filtering). This requires a user input of a lower cut-off distance (based on general prior knowledge of the shortest interatomic distance existing in the sample).

(*c*) The revision of number density. *SUePDF* is able to deduce a value of number density from the normalized ED data. If a better value of number density is known already, it should be used as the correction for the deduced value. In a good case of data processing of nanoscale samples, the deduced value can be very close to the generally accepted one.

**Figure 9:**
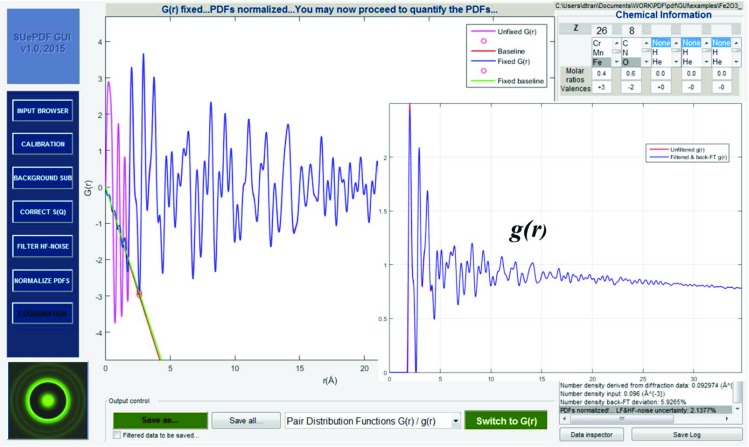
PDF renormalization: the cutting off of low-frequency distortion and number density revision; *g*(*r*) is shown in the inset.

Fig. 9 ▸ shows the PDF renormalization by cutting off low-frequency distortions and number density revision.

### 3.7. PDF quantification   

**Figure 10:**
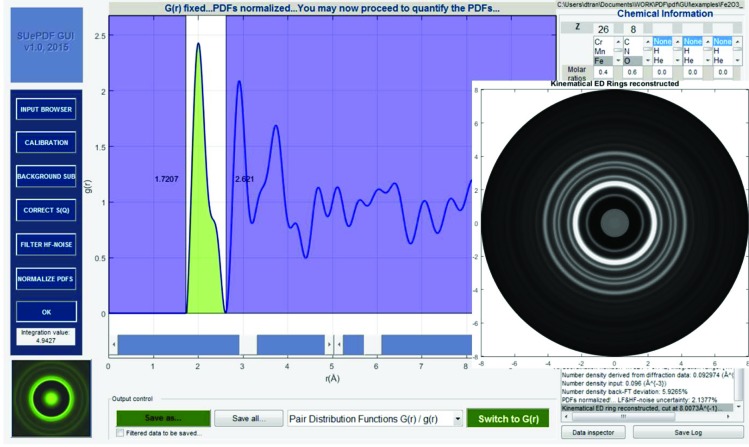
Measuring coordination number and reconstruction of the kinematical ED pattern.

As shown in Fig. 10 ▸, the coordination number can be measured by specifying an integration window for the relevant *g*(*r*) peak. The background-subtracted and noise-filtered electron scattering profile (which should be the extracted kinematical scattering data) can be reconstructed back into a ring pattern, shown as the inset of Fig. 10 ▸.

## 4. Examples   

### 4.1. Gold nanoparticles   

**Figure 11:**
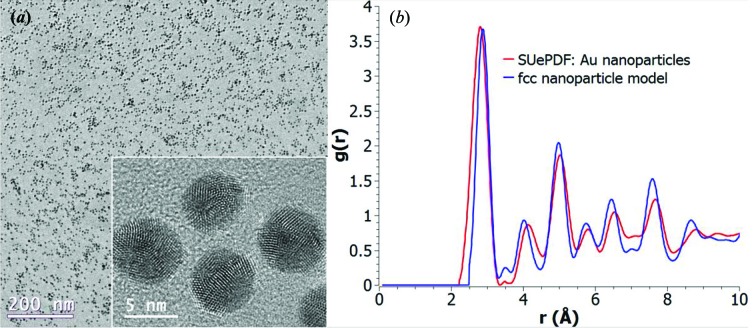
(*a*) TEM images of ∼5 nm sized Au nanoparticles including a high-resolution image (inset); (*b*) ED-based PDF obtained using *SUePDF* (red) compared with the theoretical PDF of a 5 nm spherical Au nanoparticle model having perfect fcc structure (blue).

An example of using *SUePDF* to study ∼5 nm sized Au nanoparticles supported on an amorphous carbon film is shown in Fig. 11 ▸. The particles are considered to be close to spherical in shape (thus, the nanoparticle form factor of a 5 nm sphere was chosen), although some facets resembling cuboctahedral morphology can be seen in the high-resolution transmission electron microscopy (TEM) image (inset of Fig. 11 ▸ *a*). To obtain reliable ED data for the Au nanoparticles, an ED data set of an equivalent blank carbon film was collected as the substrate reference. The PDF based on ED data with *Q*_min_ = 1.5 Å^−1^ and *Q*_max_ = 12.5 Å^−1^ is shown in Fig. 11 ▸(*b*) (red) in a comparison with the theoretical PDF of a 5 nm spherical model of a perfect face-centered cubic (fcc) Au nanoparticle (blue).

### 4.2. Amorphous silica   

**Figure 12:**
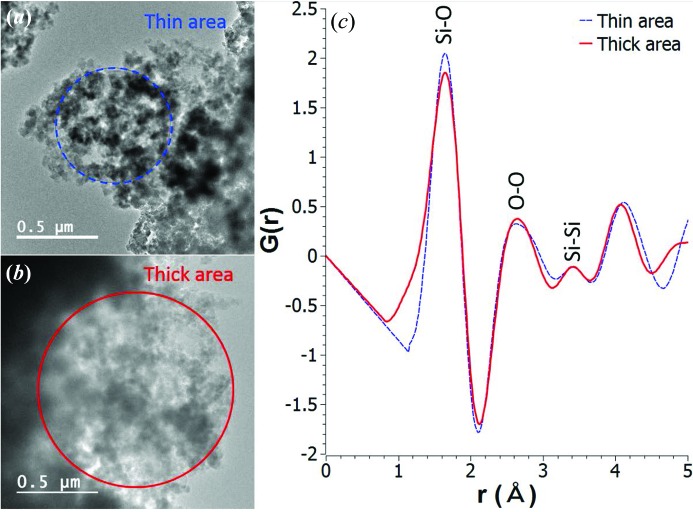
Comparison between thin and thick areas of amorphous silica for obtaining ED-based PDFs using *SUePDF*. (*a*), (*b*) TEM images of thin and thick areas, respectively, for ED acquisition; (*c*) the corresponding ED-based PDFs for the thin (dashed blue line) and thick (solid red line) areas.

Fig. 12 ▸ shows examples of amorphous silica at thin and thick areas of a sample, in order to demonstrate the validity of the background modeling for samples with different thicknesses. These areas are shown in the TEM images (Figs. 12 ▸ *a* and 12 ▸ *b*) with the selected-area apertures (marked with dashed blue and solid red circles for the thin and thick areas, respectively) defining the regions for ED acquisition. The corresponding (reduced) PDFs obtained using *SUePDF* are shown in Fig. 12 ▸(*c*). The PDF of the thin area (dashed blue line) was obtained from ED data with *Q*_min_ ≃ 0.4 Å^−1^ and *Q*_max_ ≃ 12 Å^−1^. The PDF of the thick area (solid red line) was obtained with the same *Q*_max_ but a slightly higher *Q*_min_ (0.55 Å^−1^) in order to cut off the possible increase of inelastic scattering. Note that, because of the limited *Q*_max_, the ED-based PDFs are generally broader than X-ray/neutron-based PDFs and the termination ripples may interfere significantly with some low and broad peaks of amorphous materials (*e.g.* Si—Si peaks). The quantitative measurements of bond lengths and coordination numbers for both the thin and the thick areas are listed in Table 1 ▸, together the reference data from neutron scattering (Keen & Dove, 1999 ▸) and molecular dynamics simulation of bulk amorphous silica (Hoang, 2007 ▸). The bond lengths found by *SUePDF* do not change significantly from the thin to the thick areas. On the other hand, the average coordination numbers (measured with a number density of 0.065 Å^−3^) do vary from the thin to the thick region. Apart from the possible sources of error specified in §2.5, this reasonable variance could be attributed to the finite size of the studied regions and local effects when the ED data are obtained from small amounts of sample in a transmission electron microscope, which are not large enough to be fully considered as bulk samples.

### 4.3. Metallic glass   

**Figure 13:**
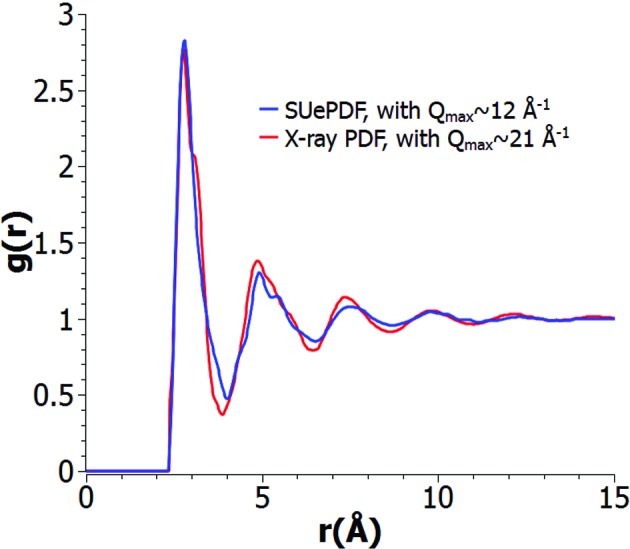
ED-based PDF (blue) of Cu_0.475_Zr_0.475_Al_0.05_ metallic glass obtained using *SUePDF* compared with the corresponding X-ray data (Kaban *et al.*, 2015 ▸) shown in red.

Fig. 13 ▸ shows an ED-based PDF of Cu_0.475_Zr_0.475_Al_0.05_ metallic glass in comparison with the corresponding X-ray data (Kaban *et al.*, 2015 ▸). The ED data have [*Q*_min_, *Q*_max_]_ED_ = [0.9, 12.2] Å^−1^, while the X-ray data have [*Q*_min_, *Q*_max_]_X_ = [0.7, 21.1] Å^−1^. Note that the difference in *Q* range can cause different termination effects on the ED-based PDF and X-ray PDF. Besides this, the sample amount in a TEM-based ED experiment is much less than the amount in an X-ray experiment, suggesting that the information given by the ED-based PDF is more local than the information given by the X-ray PDF.

## 5. Environment and distribution of *SUePDF*   

*SUePDF* is written in the MATLAB language and is compiled as a stand-alone GUI program, which only requires the free MATLAB Runtime R2015a (or newer) environment (http://www.mathworks.com) installed on a 64 bit Windows platform (Windows XP or newer is recommended). The installer (SUePDF_Installer.exe file) will automatically download (internet connection required) and install the MATLAB Runtime environment before installing *SUePDF* when executed.

*SUePDF* is distributed as free software for academic users, with an installer file and a manual document available for free download at https://osf.io/c2jq8/.

## 6. Summary   

We have described the implementation of *SUePDF*, a GUI program dedicated to structural analysis based on electron diffraction data. *SUePDF* facilitates TEM-based structural studies of amorphous materials and nanoparticles by converting the electron diffraction data in the reciprocal space into quantitative PDFs in the direct space. *SUePDF* employs the scattering physics of electrons as well as the physical meaning of PDFs to achieve reliable data normalization. Noise is treated in *SUePDF* by band-pass Fourier filtering that also allows the evaluation of uncertainties caused by experimental conditions and data treatment procedures. Examples of using *SUePDF* to obtain quantitative PDFs of crystalline Au nanoparticles, amorphous silica and amorphous Cu_0.475_Zr_0.475_Al_0.05_ metallic glass have been demonstrated.

## Figures and Tables

**Table 1:** Bond lengths and coordination numbers of amorphous silica found by using *SUePDF* on the ED data obtained from thin and thick areas These values are compared with the results from neutron (N) scattering (Keen & Dove, 1999 ▸) and molecular dynamics (MD) simulations (Hoang, 2007 ▸). Note that the first peak of the total PDF measures the average of the (Si—O) and (O—Si) coordination numbers, which is calculated as [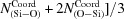.

	Bond lengths (Å)			Coordination numbers		
	Si—O	O—O	Si—Si	(Si—O) & (O—Si)	O—O	Si—Si
*SUePDF* thin area	1.63	2.62	3.40	2.49	5.49	3.89
*SUePDF* thick area	1.63	2.64	3.41	2.69	5.18	4.44
References	1.62 (N)	2.63 (N)	3.10 (N)	2.66 (MD)	6.07 (MD)	3.78 (MD)
